# Japanese encephalitis virus perturbs PML-nuclear bodies by engaging in interactions with distinct porcine PML isoforms

**DOI:** 10.3389/fcimb.2023.1239234

**Published:** 2023-10-20

**Authors:** Songbai Yang, Huaijin Liu, Zhenyu Chen, Han Wang, Xiangchen Li, Xiaolong Zhou, Ayong Zhao

**Affiliations:** Key Laboratory of Applied Technology on Green-Eco-Healthy Animal Husbandry of Zhejiang Province, College of Animal Science and Technology, College of Veterinary Medicine, Zhejiang A&F University, Hangzhou, China

**Keywords:** Japanese encephalitis virus, non-structural proteins, PML-NBs, PML isoforms, immunofluorescence analysis

## Abstract

Promyelocytic leukemia (PML) protein constitutes an indispensable element within PML-nuclear bodies (PML-NBs), playing a pivotal role in the regulation of multiple cellular functions while coordinating the innate immune response against viral invasions. Simultaneously, numerous viruses elude immune detection by targeting PML-NBs. Japanese encephalitis virus (JEV) is a flavivirus that causes Japanese encephalitis, a severe neurological disease that affects humans and animals. However, the mechanism through which JEV evades immunity via PML-NBs has been scarcely investigated. In the present study, PK15 cells were infected with JEV, and the quantity of intracellular PML-NBs was enumerated. The immunofluorescence results indicated that the number of PML-NBs was significantly reduced in JEV antigen-positive cells compared to viral antigen-negative cells. Subsequently, ten JEV proteins were cloned and transfected into PK15 cells. The results revealed that JEV non-structural proteins, NS2B, NS3, NS4A, NS4B, and NS5, significantly diminished the quantity of PML-NBs. Co-transfection was performed with the five JEV proteins and various porcine PML isoforms. The results demonstrated that NS2B colocalized with PML4 and PML5, NS4A colocalized with PML1 and PML4, NS4B colocalized with PML1, PML3, PML4, and PML5, while NS3 and NS5 interacted with all five PML isoforms. Furthermore, ectopic expression of PML isoforms confirmed that PML1, PML3, PML4, and PML5 inhibited JEV replication. These findings suggest that JEV disrupts the structure of PML-NBs through interaction with PML isoforms, potentially leading to the attenuation of the host’s antiviral immune response.

## 1 Introduction

Japanese Encephalitis Virus (JEV), classified as a single stranded RNA virus, is a member of the *Flavivirus* genus within the *Flaviviridae* family. It represents the foremost etiological agent of viral encephalitis in Asia and is primarily transmitted by mosquitoes of the *Culex tritaeniorhynchus* species (van den Hurk et al., 2009). Pigs are perceived as the principal amplifying host for the JEV. JEV can precipitate reproductive failures and induce encephalitis in swine. JEV infection in pregnant swine can cause abortion, stillbirths, and weak piglets. Piglets infected with JEV can develop non-suppurative encephalitis (Takashima et al., 1988; Yamada et al., 2004; Ricklin et al., 2016). JEV encodes a long polyprotein that undergoes post-translational processing mediated by both viral and cellular proteases. This processing leads to the generation of three structural proteins: the nucleocapsid or capsid protein (C), the non-glycosylated pre-membrane protein (prM), and the glycosylated envelope protein (E). Additionally, it produces seven non-structural proteins, namely NS1, NS2A, NS2B, NS3, NS4A, NS4B, and NS5 (Roberts and Gandhi, 2020; Alfaiz, 2022). The structural analysis of NS3 reveals that it consists of an N-terminal protease domain and a C-terminal helicase domain (Yamashita et al., 2008). These domains have been shown to be essential for the negative supercoiling of double-stranded RNA (dsRNA) intermediates during JEV replication and transcription (Warrener et al., 1993). NS5, the largest viral protein with a molecular weight of 100 kDa, is known to encompass methyltransferase (MTase) and RNA-dependent RNA polymerase (RdRp) at its N and C termini, respectively, and plays a pivotal function in the replication of JEV (Lu and Gong, 2013).

The Promyelocytic Leukemia (PML) protein, also known as TRIM19, is part of the Tripartite Motif (TRIM) family of proteins and is considered a crucial element of PML nuclear bodies (PML-NBs) (Bernardi and Pandolfi, 2007). PML nuclear bodies (PML-NBs) are dynamic structures that consist of multiple cellular proteins, including but not limited to PML, speckled protein 100 (SP100), and death domain associated protein (DAXX) (Scherer and Stamminger, 2016). PML undergoes covalent modification and co-localization by all three constituents of the SUMO family, namely SUMO1, SUMO2, and SUMO3. Throughout mitosis, de-sumoylation of PML proteins facilitates the segregation of SP100 and hDAXX from PML-NBs, whereas during interphase these proteins are enlisted and interact with each other via sumoylation to form PML-NBs (Bernardi and Pandolfi, 2007). Hence, PML-NBs participate in the regulation of numerous cellular functions, including cell cycle, apoptosis (Bernardi and Pandolfi, 2003), senescence (Bischof et al., 2002), stress (Maul et al., 1995), and DNA damage response (Yang et al., 2002; Bernardi et al., 2004). Early studies have demonstrated that PML is a tumor suppressor (Salomoni and Pandolfi, 2002). Recent studies have also revealed the antiviral impact of PML-NBs. PML-NBs are capable of impeding viral replication by ensnaring the viral genome and inhibiting viral gene expression (Scherer and Stamminger, 2016). PML and PML-NBs have been demonstrated to inhibit the replication of diverse viruses, including herpes simplex virus 1 (HSV-1) (Cuchet et al., 2011), dengue virus (DENV) (Giovannoni et al., 2015), influenza A virus (IAV) (Yan et al., 2021), human cytomegalovirus (HCMV) (Tavalai et al., 2006), human immunodeficiency virus 1 (HIV-1) (Kahle et al., 2015). Simultaneously, PML-NBs interact with Interferon-stimulated genes (ISGs) and the Signal Transducer and Activator of Transcription (STAT) to participate in the regulation of innate immunity (Chen et al., 2015).

In response to the antiviral effects of PML-NBs, various viral proteins dismantle PML-NBs by degrading their components to counteract the antiviral activity (Scherer and Stamminger, 2016). For instance, HSV-1 infected cell protein 0 (ICP0) is capable of disassembling PML-NBs via the degradation of PML-NB components like PML to counteract the antiviral response (Boutell et al., 2011). HCMV encodes a protein known as IE1 that has the ability to disrupt PML-NBs. IE1 has been demonstrated to induce a loss of SUMO-modified PML, a crucial step in the formation and functionality of PML-NBs (Scherer et al., 2017). Likewise, the DENV NS5 protein disintegrates PML-NBs by forming complexes with PML3 and PML4 isoforms (Giovannoni et al., 2019). Nonetheless, the process by which the JEV impedes PML-NBs remains unclear.

We previously identified seven alternative splicing variants of porcine PML that encode five proteins and examined the expression of these five PML isoforms during JEV infection (Zhu et al., 2021). In this study, we investigated how JEV counteracts the structure of PML-NBs via its expressed proteins. The results showed a decrease in the count of intracellular PML-NBs following JEV infection. JEV NS2B, NS3, NS4A, NS4B, and NS5 significantly diminished the quantity of PML-NBs. Furthermore, confocal microscopy results demonstrated that both NS3 and NS5 interacted with PML 1-5 isoforms. Overexpression of PML1, PML3, PML4, and PML5 has shown that they inhibit JEV replication. These findings suggest that JEV disrupts the structure of PML-NBs through interaction with PML isoforms, potentially leading to the attenuation of the host’s antiviral immune response.

## 2 Materials and methods

### 2.1 Cell culture

PK15 (porcine kidney) and BHK-21 (baby hamster kidney) cells, obtained from the China Center for Type Culture Collection (CCTCC, Wuhan, China), were cultured in Minimal Essential Medium (MEM; HyClone, Logan, UT, USA) supplemented with 10% fetal bovine serum (FBS; HyClone) and an additional 1% non-essential amino acids (Gibco-BRL Life Technologies, Grand Island, NY, USA). The cells were incubated at 37°C with a 5% CO_2_ atmosphere.

### 2.2 Virus infection

PK15 cells were grown in 12-well culture plates and then infected with JEV strain SA14-14-2 (MOI = 1) (Yang et al., 2016), diluted in MEM. Following a 1h adsorption period, the cells were washed 3 times with PBS. Subsequently, the cells were subjected to a continuous 36h infection period in MEM supplemented with 2% FBS.

### 2.3 Plasmid construction

The open reading frame (ORF) of 10 JEV viral proteins (C, prM, E, NS1, NS2A, NS2B, NS3, NS4A, NS4B, NS5) were amplified using specific forward and reverse primers listed in **Table 1**. These amplified sequences were then ligated to the pmCherry-N1 vector (Clontech, Mountain View, CA, USA) to generate the Cherry-C, prM, E, NS1, NS2A, NS2B, NS3, NS4A, NS4B, NS5 expression vectors. Additionally, EGFP-PML1, PML2, PML3, PML4, and PML5 vectors were provided by our laboratory (Zhu et al., 2021). The pcDNA3.1 vector (Invitrogen, Carlsbad, CA, USA) expression plasmids for the PML isoforms were constructed using recombinant pEGFP-C1 plasmids.

**Table 1:** The primer sequences used for the construction of gene expression vectors.

Names	Primer sequence (5’-3’)
C	F: CCGCTCGAGATGACTAAAAAACCAGGAGG
	R: CCCAAGCTTTCTTTTGTTTTGCTTTCTGC
prM	F: CCGCTCGAGATGAAGTTGTCGAATTTCCA
	R: CGGGATCCCGACTGTAAGCCGGAGCGACCA
E	F: CCGCTCGAGATGTTTAATTGTCTGGGAATGGG
	R: CCCAAGCTTCACATTGGTCGCTAAGAACA
NS1	F: CCGCTCGAGATGGACACTGGATGTGCCATTGA
	R: CCCAAGCTTAGCATGAACCTGTGATCTG
NS2a	F: CCGCTCGAGATGTTCAAAGGTGAAATGGTTGA
	R: CCCAAGCTTTCTCTTCTTGTTTGGGTTGC
NS2b	F: AAACTCGAGATGGGGTGGCCAGCTACTGA
	R: GGGAAGCTTTCTTTTTGTTGTTTTTAAAG
NS3	F: AAACTCGAGATGGGGGGCGTGTTTTGGGACAC
	R: GGAATTCCTCTCTTCCCTGCTGCAAAGT
NS4a	R: CCGCTCGAGATGTCAGCCGTTAGCTTCATAGAGG
	R: CGGGATCCCGCCTCTGTTTTTCCGGTTCTGG
NS4b	F: CCGCTCGAGATGAACGAGTACGGGATGCTAGA
	R: CCCAAGCTTCCTTTTCAAGGAGGGCTTAT
NS5	F: CCGCTCGAGATGGGAAGGCCTGGGGGCA
	R: CGGGATCCCGGATGACCCTGTCTTCCTG

F, forward primer; R, reverse primer. The enzyme cleavage site was underlined.

### 2.4 Immunofluorescence analysis

Cells were cultured on cover slides placed in 24-well plates and subsequently infected with JEV or transfected with the respective expression vectors when they reached approximately 80% confluence. The cells were subsequently subjected to immunofluorescence analysis at the indicated hours post-transfection. Briefly, the cells were fixed with 300 μL 4% paraformaldehyde on ice for 15 min. Then, the cells were permeabilized with 300 μL of 0.25% Triton X-100 on ice for 10 min. After that, the cells were washed three times with PBS and then blocked with 200μL of PBS containing 10% FBS and 3% BSA for 2h. The cells were incubated with a rabbit anti-PML antibody (1:100, GeneCreate, China) or a mouse monoclonal anti-JEV E antibody (1:20, ab41671, Abcam, Cambridge, MA, USA) overnight at 4°C. Following three PBS washes, the cells were then incubated for 2h with Alexa Fluor 488-conjugated goat anti-rabbit antibody (diluted at 1:1000; A-11008, Invitrogen, Carlsbad, CA, USA) or Alexa Fluor 555-conjugated goat anti-mouse antibody (A-21422, Invitrogen), ensuring light avoidance. The nuclei were then stained with DAPI (Beyotime Biotechnology, Shanghai, China) for 10 min. Fluorescence images were acquired with a laser scanning confocal microscope (FV3000, Olympus, Japan). Image processing and analysis were performed with Fiji software (http://fiji.sc). The analysis of colocalization was performed using the Fiji Colocalization Finder Plugin.

### 2.5 Quantitative reverse transcription polymerase chain reaction

PK15 cells were seeded into 12-well plates. When the cells reached approximately 80% confluency, they were either treated with various concentrations of IFN-β (0, 10, 100, 500ng/mL; 300-02BC, PeproTech, Rocky Hill, NJ, USA) for 24h. Alternatively, the cells were transfected with different overexpression vectors of PML isoforms for 36h or infected with JEV for an additional 36h. Total RNA was isolated using TRIzol reagent (Invitrogen), and the concentration and quality total RNA were assessed using a Nano300 ultra-micro spectrophotometer (Allsheng Instruments, Hangzhou, China). A total of 1 μg of total RNA was utilized to synthesize complementary DNA (cDNA) using a 5X All-In-One RT MasterMix kit (ABM, Vancouver, BC, Canada). Quantitative analysis was performed to assess the expression levels of PML mRNA using SYBR Premix Ex Taq II (Takara, Dalian, China) on a CFX96 Touch instrument (Bio-Rad, Richmond, California, USA). The sequences of the qPCR primers used are as follows: PML-Forward: CGGAAGGAAGCCAAATGC, PML-Reverse: TATCCAGGGCCTGCGTGT. JEV-E-Forward: GTCCATAGGGAGTGGTTTCA, JEV-E-Reverse: CCTTTCAGAGCCAGTTTGTC. GAPDH-Forward: GGACTCATGACCACGGTCCAT, GAPDH-Reverse: TCAGATCCACAACCGACACGT. The PCR cycling conditions used were as follows: initial denaturation at 95°C for 2 minutes, followed by 40 cycles of denaturation at 95°C for 5 seconds, annealing at 60°C for 30 seconds, and extension at 72°C for 20 seconds. Subsequently, melting curve analysis was performed. The relative expression levels were calculated using the 2^-ΔΔCt^ method (Livak and Schmittgen, 2001), with GAPDH serving as a reference gene.

### 2.6 CCK-8 assay

Cell viability was assessed using the Cell Counting Kit-8 (CCK-8) assay kit (C0037; Beyotime, Shanghai, China). Briefly, PK15 cells were treated with various concentrations of IFN-β (0, 10, 100, 500ng/mL) for 24h and subsequently washed three times with PBS. Following that, the cells were incubated with a 10% dilution of CCK-8 reagent in fresh MEM for 4 h at 37°C. The absorbance at 450nm was measured using an iMark microplate absorbance reader (Bio-Rad).

### 2.7 Western blot analysis

Protein isolation was carried out using RIPA buffer supplemented with phosphatase and protease inhibitors (CoWin Biosciences). Proteins were separated via sodium dodecyl sulfate-polyacrylamide gel electrophoresis (SDS-PAGE) and subsequently transferred onto polyvinylidene difluoride (PVDF) membranes. The PVDF membranes were blocked for 1h using a solution that consisted of 5% nonfat milk diluted in Tris-buffered saline with 0.1% Tween-20 (TBST). Afterward, they were incubated overnight at 4°C with primary antibodies for NS3 (GTX125868, GeneTex) or β-actin (20536-1-AP, Proteintech). Following this, the membranes were subjected to three TBST washes before being incubated with HRP-conjugated secondary antibodies at room temperature for 1h. Lastly, protein band visualization was performed using a chemiluminescent HRP substrate (Merck Millipore, Darmstadt, Germany). Protein band imaging was conducted using the Tanon 5200 system (Tanon Science and Technology, Shanghai, China).

### 2.8 Statistical analysis

The data presented are the mean ± SEM of three independent experiments. Statistical significance was assessed using Student’s t-test for two groups and one-way analysis of variance (ANOVA) followed by Dunnett’s test for multiple groups, using GraphPad Prism software. Statistical significance was denoted as follows: * *P* < 0.05, ** *P* < 0.01, *** *P* < 0.001, and **** *P* < 0.0001.

## 3 Results

### 3.1 The number of PML-NBs was reduced in JEV antigen-positive cells

**Figure 1:**
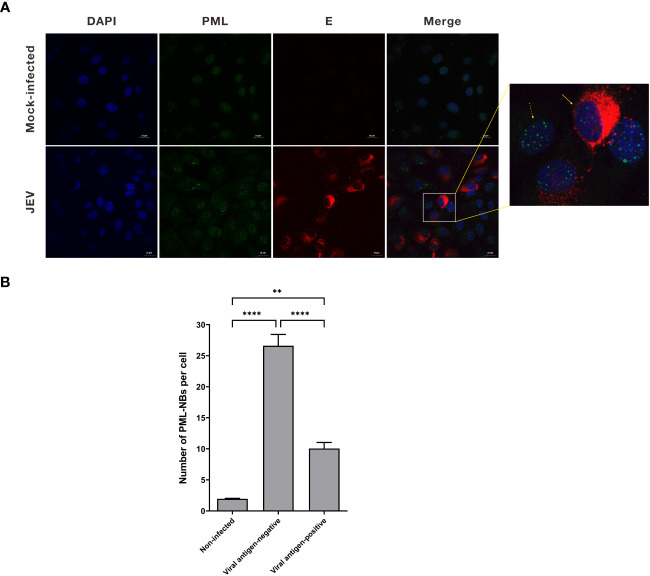
The number of PML-NBs was reduced in JEV antigen-positive cells. **(A)** PK15 cells were infected with JEV for 36h. Immunofluorescent staining was conducted to visualize the PML-NBs using an anti-PML antibody (green), and to detect JEV using an anti-JEV E antibody (red). The nuclei were stained with DAPI. The solid arrow represents viral antigen-positive cell, while the dotted arrow represents viral antigen-negative cell. **(B)** The average count of PML-NBs per nucleus was determined using Fiji software, with a minimum of 40 cells per condition being analyzed. The scale bar represents 20μm. ** *P* < 0.01, **** *P* < 0.0001.

PML-NBs have been demonstrated to suppress the replication of various viruses. However, viral proteins can disrupt the structure of PML-NBs through multiple mechanisms to counteract their antiviral activity (Scherer and Stamminger, 2016). To investigate the potential impact of JEV on the number of PML-NBs in cells, PK15 cells were infected with JEV for 36h. Subsequently, the cells were subjected to immunofluorescent staining to visualize nuclear bodies (NBs) using a PML antibody, as well as JEV using a JEV E antibody. The red channel for JEV and the green channel for PML were analyzed using ImageJ software. This allowed us to quantify PML-NBs in both viral antigen-positive and viral antigen-negative cells. Double immunofluorescence studies indicated a significant decrease in the number of PML-NBs in the majority of viral antigen-positive cells compared to viral antigen-negative cells. In uninfected cells, the number of PML-NBs was relatively low, approximately 2-3 PML-NBs per cell. However, in cells exposed to the JEV virus, the number of PML-NBs in viral antigen-negative cells increased to about 27. In contrast, in viral antigen-positive cells, the number of PML-NBs significantly decreased to roughly 10 per cell, compared to viral antigen-negative cells (**Figures 1A, B**). These results suggest that the number of PML-NBs was significantly reduced in JEV antigen-positive cells compared to viral antigen-negative cells, indicating that the protein expressed by the JEV can disrupt the structure of PML-NBs.

### 3.2 Treatment with IFN-β leads to an increase in the number of PML-NBs

**Figure 2:**
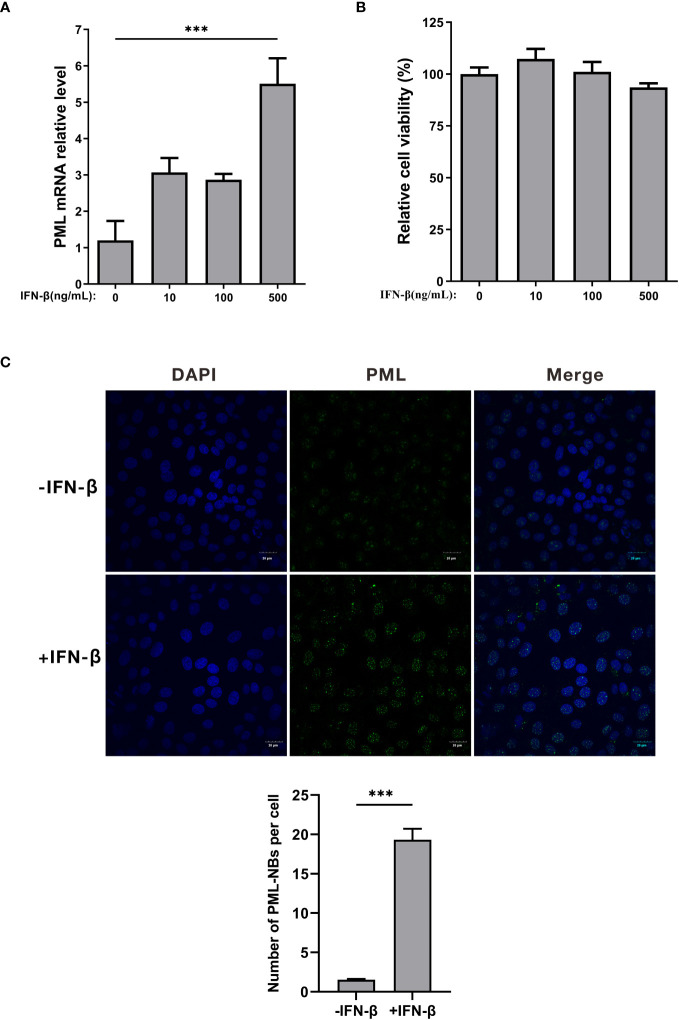
IFN-β treatment increases the number of PML-NBs. **(A)** PK15 cells were treated with varying concentrations of IFN-β (0, 10, 100, 500ng/mL) for 24h. PML mRNA expression levels in each group were determined using qRT-PCR. **(B)** Cell viability was assessed using the CCK-8 assay after treatment with varying concentrations of IFN-β. **(C)** PK15 cells were treated with 500ng/mL IFN-β for 24h. Immunofluorescence staining was conducted using an anti-PML antibody. Nuclear staining was performed with DAPI, and the cells were then observed under confocal microscopy. The average count of PML-NBs per nucleus was determined using Fiji software, with at least 25 cells from each condition being counted and analyzed. The scale bar corresponds to 20μm. *** *P* < 0.001.

Previous studies have demonstrated that the expression of the PML protein is stimulated by interferon (IFN). Furthermore, IFN treatment has been shown to enhance the formation of PML-NBs (Regad and Chelbi-Alix, 2001). To verify whether the number of PML-NBs is regulated by IFN in PK15 cells, PK15 cells were treated with varying concentrations of IFN-β (0, 10, 100, 500ng/mL) for 24h. The expression level of PML mRNA in the cells was detected using the qRT-PCR technique (**Figure 2A**). The results indicated a significant increase in PML mRNA levels after treatment with 500ng/mL of IFN-β, compared to the control group (**Figure 2A**). Additionally, the cellular activities in the IFN-β-treated group did not exhibit a significant change when compared to the control group (**Figure 2B**). Accordingly, PK15 cells were treated with 500ng/mL of IFN-β for 24h. Subsequently, the cells were stained using PML antibody for immunofluorescence, and the number of NBs was counted. In the IFN-treated group, the average number of PML-NBs per cell was 18, which was significantly higher compared to the average of 2 in the control group (**Figure 2C**). These results demonstrated that treating PK15 cells with IFN-β could increase the number of PML-NBs within the cells.

### 3.3 JEV NS2B, NS3, NS4A, NS4B, and NS5 reduce the number of PML-NBs

**Figure 3:**
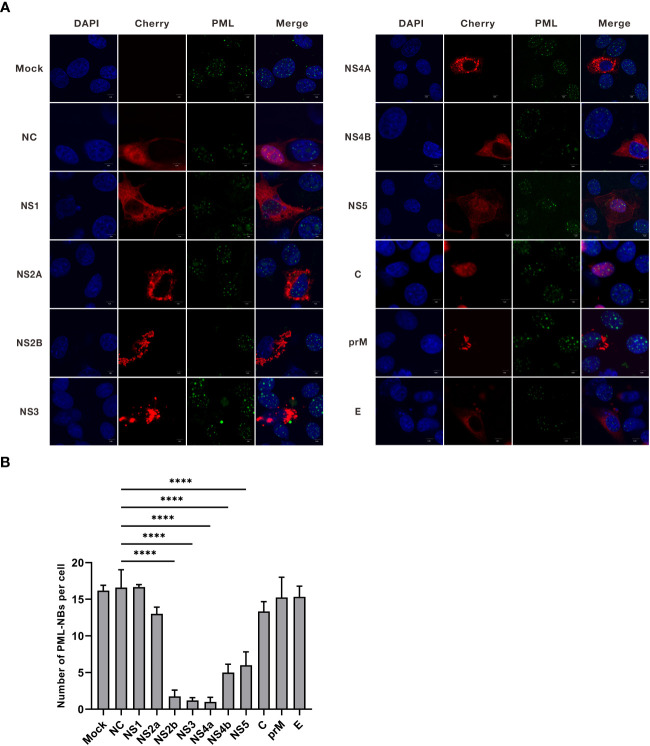
The nonstructural proteins of JEV, NS2b, NS3, NS4a, NS4b, and NS5, have been shown to reduce the number of PML-NBs. **(A)** Ten overexpression vectors, including Cherry-NS1, NS2A, NS2B, NS3, NS4A, NS4B, NS5, C, prM, and E, were individually transfected into PK15 cells. Twenty-four hours post-transfection, the cells were treated with an additional 500 ng/mL of IFN-β for another 24h. The cells were then fixed and subjected to immunofluorescence using an anti-PML antibody, and nuclear staining was carried out using DAPI. Images were captured using confocal microscopy. ‘Mock’ represents an image from the group that underwent IFN-β treatment but not transfection. ‘NC’ represents the group that received empty vector transfection. **(B)** The number of PML-NBs in each group of cells was counted and analyzed using Fiji software, with at least 15 cells per condition. The scale bar corresponds to 5μm. **** *P* < 0.0001.

JEV encodes three structural proteins and seven nonstructural proteins (Zhu et al., 2023). To investigate the mechanism by which JEV disrupts PML-NBs, we generated overexpression vectors for JEV proteins, including Cherry-NS1, NS2A, NS2B, NS3, NS4A, NS4B, NS5, C, prM, and E. Subsequently, these 10 overexpression vectors were individually transfected into PK15 cells. Twenty-four hours post-transfection, the cells were treated with 500ng/mL of IFN-β for an additional 24h. The cells were then collected and subjected to immunofluorescence (**Figure 3A**). Compared to the empty vector control group (NC), the average number of PML-NBs in cells transfected with NS2B, NS3, and NS4A decreased from 17 to 2. Similarly, the average number of PML-NBs in cells transfected with NS4b and NS5 decreased to 5 (**Figure 3B**). These findings indicate that JEV NS2B, NS3, NS4A, NS4B, and NS5 contribute to the reduction in the number of PML-NBs.

### 3.4 JEV NS2B, NS3, NS4A, NS4B, and NS5 colocalize with different PML isoforms

**Figure 4:**
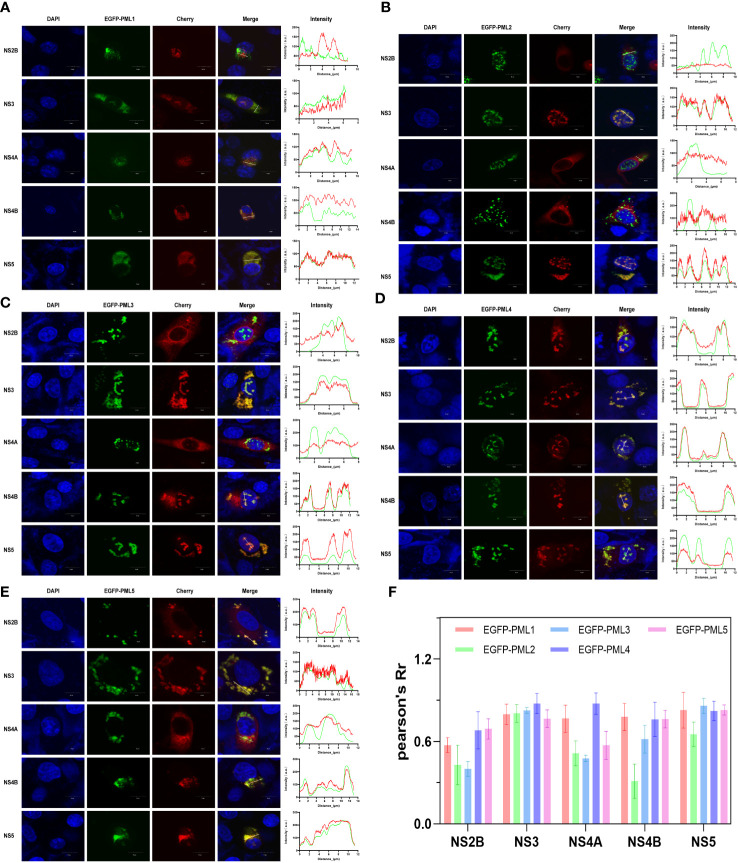
JEV NS2B, NS3, NS4A, NS4B, and NS5 exhibit colocalization with distinct PML isoforms. BHK-21 cells were co-transfected with one of the PML isoforms (PML1-5) and each of the five viral proteins: NS2B, NS3, NS4A, NS4B, and NS5. In the overexpression vector, the PML isoform was fused with green fluorescent proteins (GFP), and the viral protein was fused with red fluorescent protein (mCherry). After 24h of transfection, the cells were collected and fixed, followed by nuclear staining with DAPI. The colocalization of PML isoforms and viral proteins was then observed under a confocal microscope. The colocalization of each PML isoform- **(A)** PML1, **(B)** PML2, **(C)** PML3, **(D)** PML4, and **(E)** PML5- with each of the five viral proteins. The line charts display the fluorescence intensity values of PML isoforms (green) and viral proteins (red), as determined along the path of the white line depicted in the corresponding inset panel. The scale bar represents 10μm. **(F)** Quantitative analysis of the co-localization of 5 PML isoforms and 5 viral proteins using Fiji software.

We previously identified five alternative splice forms of the porcine PML gene, which we named as PML1, PML2, PML3, PML4, and PML5 (Zhu et al., 2021). To identify which of the five porcine PML isoforms could potentially recruit JEV NS2B, NS3, NS4A, NS4B, and NS5 proteins into PML-NBs. The overexpression vector for each PML isoform was fused with green fluorescent protein (eGFP) and co-transfected with the overexpression vector of each of the five viral proteins. Conversely, the overexpression vector for each viral protein was fused with red fluorescent protein (mCherry). After 24 hours of co-transfection, immunofluorescence staining was performed, and the colocalization of PML isoforms and viral proteins was observed under a confocal microscope. The results obtained from confocal microscopy were analyzed using ImageJ software (**Figure 4**). The findings revealed that the PML1 isoform colocalizes with JEV NS3, NS4A, NS4B, and NS5 proteins (**Figure 4A**). The PML2 isoform colocalizes with JEV NS3 and NS5 proteins (**Figure 4B**). The PML3 isoform colocalizes with JEV NS3, NS4B, and NS5 proteins (**Figure 4C**). The PML4 isoform colocalizes with JEV NS2B, NS3, NS4A, NS4B and NS5 proteins (**Figure 4D**). The PML5 isoform colocalizes with JEV NS2B, NS3, NS4B, and NS5 proteins (**Figure 4E**). Pearson’s coefficient serves as a colocalization coefficient for two proteins. If Pearson’s R value is greater than 0.6, it is considered that there is colocalization between the two proteins. This suggests that NS2B colocalizes with PML4 and PML5, NS4A colocalizes with PML1 and PML4, NS4B colocalizes with PML1, PML3, PML4, and PML5, while NS3 and NS5 colocalize with all five PML isoforms (**Figure 4F**).

### 3.5 Effect of overexpression of PML isoforms on JEV replication

**Figure 5:**
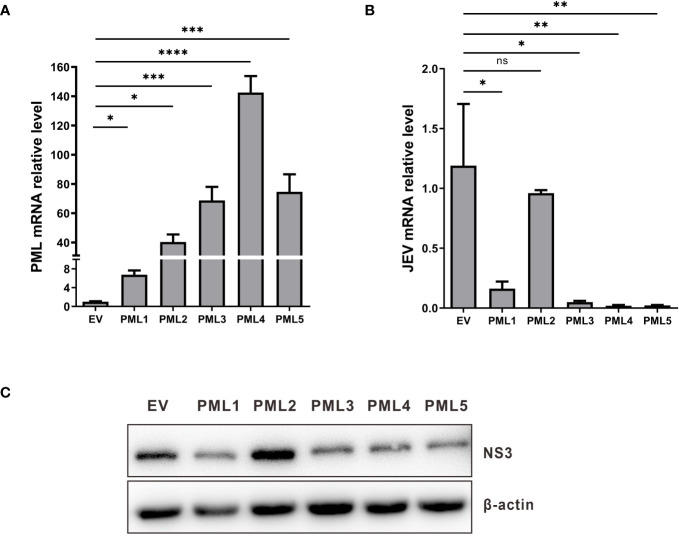
PML1, PML3, PML4, and PML5 suppress JEV replication. **(A)** pcDNA3.1-PML1, pcDNA3.1-PML2, pcDNA3.1-PML3, pcDNA3.1-PML4, pcDNA3.1-PML5, and an empty vector (EV) were transfected into PK15 cells, respectively. After 36 hours of transfection, the expression levels of PML were examined by qRT-PCR. **(B)** The PML overexpression vectors were transfected into PK15 cells for 36 h, followed by infection with JEV. Cells were collected after 36h of infection, and JEV mRNA expression was assessed using qRT-PCR. **(C)** NS3 protein expression was evaluated through Western blot analysis. *P < 0.05, **P < 0.01, ***P < 0.001, and ****P < 0.0001, ns: no significance.

PML isoforms have the capability to suppress the replication of various viruses. To verify the influence of various porcine PML isoforms on JEV replication. The overexpression vectors for PML isoforms, including pcDNA3.1-PML1, pcDNA3.1-PML2, pcDNA3.1-PML3, pcDNA3.1-PML4, pcDNA3.1-PML5, and an empty vector, were individually transfected into PK15 cells. After 36 hours of transfection, the cells were infected with JEV for another 36h. The viral genomic mRNA and protein expression were assessed using qRT-PCR and Western blot. The results indicate that, compared to the empty vector control group, the expression levels of PML in the group transfected with PML isoform overexpression vectors were significantly increased (**Figure 5A**). In the PML1, PML3, PML4, and PML5 overexpression groups, the expression levels of JEV genomic mRNA (**Figure 5B**) and NS3 protein (**Figure 5C**) were significantly decreased. These results demonstrate that PML1, PML3, PML4, and PML5 could suppress JEV replication.

## 4 Discussion

Viral replication encompasses binding, endocytosis, decapsidation, translation, assembly, maturation, and release (Yun and Lee, 2018). Upon invasion into the host cell, JEV initially undergoes translation in the cytoplasm to generate a polyprotein which can be cleaved by host and viral proteases to produce structural proteins and non-structural proteins. Structural proteins are incorporated into the viral particle, while non-structural proteins participate in the formation of the replication complex (RC) and assembly complex (AC), and replication of viral genomic RNA (Kumar et al., 2022). There are several studies concerning immune evasion by RNA viruses through the degradation of PML nuclear bodies (PML-NBs). Members of the Flavivirus family, such as the Zika Virus (ZIKV) and DENV, have been shown to reduce the count of PML-NBs in infected cells during viral infection (Giovannoni et al., 2015; García et al., 2020). Through IF analysis utilizing anti-PML antibody to stain the PML-NBs, we previously observed that the number of PML-NBs increased following JEV infection. To investigate whether the JEV, also a member of the Flavivirus family, can diminish the count of PML nuclear bodies (PML-NBs) in cells. Following virus infection, we utilized JEV E protein antibody and PML antibody for dual staining and confocal laser photography, subsequently counting the number of PML-NBs in JEV antigen-positive cells and viral antigen-negative cells. Similar to the outcomes observed in ZIKV and DENV-infected cells, our results indicate that JEV infection may reduce the number of PML-NBs in viral antigen-positive cells compared to viral antigen-negative cells. The number of intracellular PML-NBs in the control group is relatively low, with an average of 2 PML-NBs per cell. This could be attributed to the frequent loss of PML protein expression in certain cancer cells, leading to a significantly lower count of PML-NBs in the PK15 cell lines compared to primary porcine kidney cells (Gurrieri et al., 2004; Yu et al., 2020). In the infected group, viral antigen-negative cells exhibited a significantly higher quantity of PML-NBs compared to the cells in the uninfected group. This is consistent with our previous findings and the results observed in DENV-infected cells (Giovannoni et al., 2015; Zhu et al., 2021). A plausible explanation for this might be that the IFN secreted by JEV-infected cells triggers an increase in the number of PML-NBs in the adjacent cells (Giovannoni et al., 2015). Given the low count of PML-NBs in PK15 cells, we used IFN to treat these cells for subsequent experiments. We found a significant increase in the expression of PML genes and the number of PML-NBs post-IFN treatment. This finding aligns with the characterization of PML as an IFN-induced gene (Scherer and Stamminger, 2016).

Viral proteins counteract the antiviral effects of PML-NBs by disassembling these structures and breaking down their components in response to their antiviral activity (Scherer and Stamminger, 2016). We separately transfected PK15 cells with each of ten different JEV proteins to investigate their respective effects on the count of PML-NBs. The results demonstrated that JEV NS2B, NS3, NS4A, NS4B and NS5 proteins resulted in a significant decrease in the number of PML-NBs in cells. NS2B protein is the smallest non-structural protein, and it interacts with the N-terminal protease structural domain of NS3 protein to form the NS2B-NS3 protease complex, in which NS2B acts as a cofactor (Yusof et al., 2000). The NS2B-NS3 protease complex is involved in the hydrolysis and modification of the multiprotein after JEV replication. During JEV replication and transcription, the C-terminal decapping enzyme and NTPase structural domains of NS3 are required for negative supercoiling of dsRNA intermediates (Warrener et al., 1993). NS4A regulates the NTPase activity of NS3 helicase (Shiryaev et al., 2009), while NS4b is a cofactor for NS3 helicase activity (Zou et al., 2015). NS5, being the largest viral protein, contains both a methyltransferase structural domain at its N terminus and an RdRp structural domain at its C terminus (Lu and Gong, 2013). The RdRp structural domain of NS5 consists of three sub-structural domains: palm, thumb, and finger. These domains play crucial roles in the respective biological processes of viral RNA binding, transfer, and synthesis (Ng et al., 2008), and play a central role in viral replication. The results reveal that these specific JEV proteins collaboratively disrupt the antiviral functions of PML-NBs. This activity plays a pivotal role in facilitating the replication of the virus within host cells.

In our previous work, we identified seven alternative splicing variants of porcine PML. While all these variants share the same N-terminal sequence, they exhibit differences in their C-terminal sequences (Zhu et al., 2021). Each PML isoform has a unique C-terminal sequence, which results in distinct roles for each PML isoform within the cell (Nisole et al., 2013). PML II was shown to positively regulate inducible gene expression during the type I IFN response (Meng et al., 2021). PML II has also been shown to repress viral gene transcription during pseudorabies virus (PRV) infection (Yu et al., 2020). During DENV infection, the association of NS5 with PML III/IV promotes the disassembly of PML-NBs, thereby facilitating immune escape (Giovannoni et al., 2019). In this study, confocal microscopy analysis revealed that JEV NS2B, NS3, NS4A, NS4B, and NS5 co-localized with two or more of the five isoforms of porcine PML. Among them, PML1 co-localized with NS3, NS4A, NS4B, and NS5. PML3 co-localized with NS3, NS4B, and NS5. PML4 co-localized with all five viral proteins individually. PML5 co-localized with NS2B, NS3, NS4B, and NS5. However, PML2 co-localized with NS3 and NS5, and not with NS2B, NS4A, or NS4B. Moreover, the overexpression results demonstrated that PML1, PML3, PML4, and PML5 inhibited JEV replication. These results indicate that these five viral proteins are likely to utilize PML1, PML3, PML4, and PML5 isoforms to suppress the antiviral immune response. Notably, NS3 and NS5 demonstrated relatively strong co-localization with all five PML isoforms. This suggests that two vital proteins, NS3 and NS5, which are involved in viral replication, play a crucial role in antagonizing the generation of PML-NBs and suppressing the immune response. Furthermore, the interaction between the viral proteins NS2B, NS3, NS4A, NS4B, and NS5 and the PML isoforms can be further confirmed using the co-immunoprecipitation (Co-IP) method. This could help identify key domains of their interaction and provide new targets for drug development.

## 5 Conclusion

This study elucidates the potential of JEV to interfere with PML-NBs. Upon infection of PK15 cells with JEV, a significant reduction in the number of PML-NBs was observed. Subsequent investigation revealed that the non-structural proteins of JEV, specifically NS2B, NS3, NS4A, NS4B, and NS5, each have the capacity to disrupt PML-NBs. Further analysis revealed that these five viral proteins can co-localize with different PML isoforms, suggesting a mechanism whereby JEV disrupts the structure of PML-NBs through these interactions. These findings provide valuable insights into the potential strategies employed by JEV to subvert the host’s antiviral immune response.

## Data availability statement

The raw data supporting the conclusions of this article will be made available by the authors, without undue reservation.

## Author contributions

SY designed the experiments and wrote the manuscript. HL performed most of the experiments and wrote the manuscript. ZC participated in some of the experiments. HW and XL participated in data analysis. XZ and AZ contributed to the study design. All authors contributed to the article and approved the submitted version.
